# 4,5-Diiodo-2-phenyl-1*H*-imidazole

**DOI:** 10.1107/S1600536812003017

**Published:** 2012-01-31

**Authors:** Tomáš Chlupatý, Patrik Pařík, Zdeňka Padělková

**Affiliations:** a Department of General and Inorganic Chemistry, Faculty of Chemical Technology, University of Pardubice, Studentská 573, 53210 Pardubice, Czech Republic; b Institute of Organic Chemistry and Technology, Faculty of Chemical Technology, University of Pardubice, Studentská 573, 53210 Pardubice, Czech Republic

## Abstract

The structure of the title compound, C_9_H_6_I_2_N_2_, contains two symmetry-independent mol­ecules. The inter­planar angles between the imidazole and phenyl ring planes are 16.35 (3) and 17.48 (6)°. Mol­ecules are connected *via* N—H⋯N hydrogen bonds to form zigzag chains along the *b* axis. The title compound is the first example of a structurally characterized 4,5-diiodo­imidazole with an organic substituent in the 2-position and without protection on the N—H group of imidazole.

## Related literature

For the structures of various related compounds, see: Delest *et al.* (2008 ▶); Poverlein *et al.* (2007 ▶); Panday *et al.* (2000 ▶); Phillips *et al.* (1997 ▶); Terinek & Vasella (2003 ▶); Mukai & Nishikawa (2010*a* ▶,*b* ▶); Noland *et al.* (2003 ▶); Dou & Weiss (1992 ▶); Nagatomo *et al.* (1995 ▶). For the use of diiodo­imidazoles as starting compounds for ligand synthesis, see: Haruki *et al.* (1965 ▶); Ito & Uedaira (2004 ▶); Kim *et al.* (1999 ▶); Zhang *et al.* (2006 ▶). For the synthetic procedure, see: Garden *et al.* (2001 ▶); Ishihara & Togo (2006 ▶). For typical bond lengths, see: Allen *et al.* (1987 ▶).
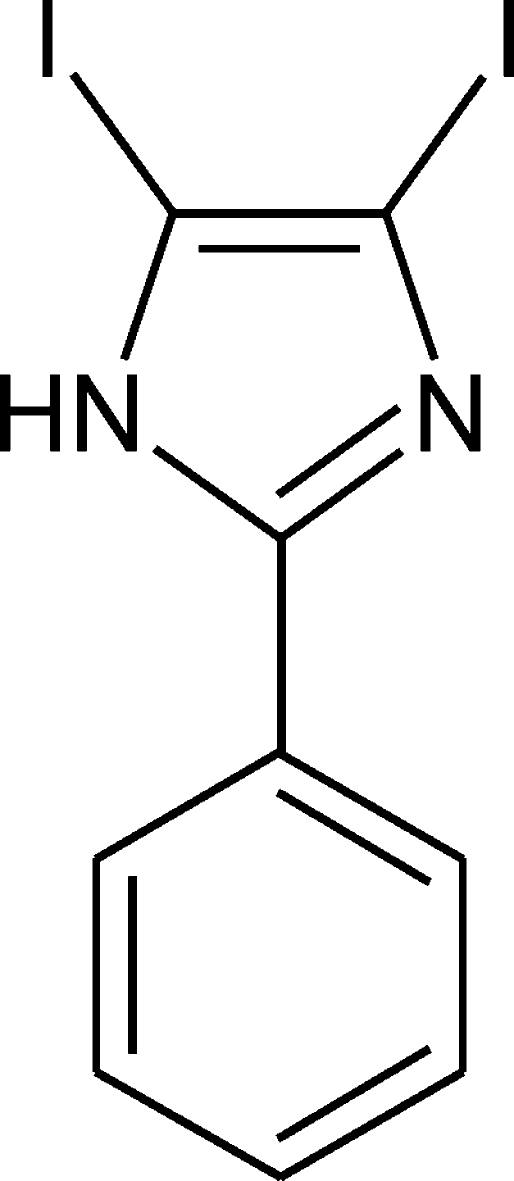


## Experimental

### 

#### Crystal data

C_9_H_6_I_2_N_2_
*M* *_r_* = 395.96
Orthorhombic, 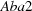

*a* = 31.0150 (6) Å
*b* = 17.5010 (5) Å
*c* = 8.0461 (9) Å
*V* = 4367.4 (5) Å^3^
*Z* = 16
Mo *K*α radiation
μ = 5.72 mm^−1^
*T* = 150 K
0.45 × 0.16 × 0.07 mm

#### Data collection

Bruker–Nonius KappaCCD area-detector diffractometer
Absorption correction: gaussian (Coppens, 1970 ▶) *T*_min_ = 0.256, *T*_max_ = 0.675
20468 measured reflections
4914 independent reflections
4499 reflections with *I* > 2σ(*I*)
*R*_int_ = 0.058

#### Refinement

*R*[*F* ^2^ > 2σ(*F* ^2^)] = 0.029
*wR*(*F* ^2^) = 0.051
*S* = 1.04
4914 reflections
235 parameters
1 restraint
H-atom parameters constrained
Δρ_max_ = 0.63 e Å^−3^
Δρ_min_ = −0.63 e Å^−3^
Absolute structure: Flack (1983 ▶), 2233 Friedel pairs
Flack parameter: 0.01 (3)

### 

Data collection: *COLLECT* (Hooft, 1998 ▶) and *DENZO* (Otwinowski & Minor, 1997 ▶); cell refinement: *COLLECT* and *DENZO*; data reduction: *COLLECT* and *DENZO*; program(s) used to solve structure: *SIR92* (Altomare *et al.*, 1994 ▶); program(s) used to refine structure: *SHELXL97* (Sheldrick, 2008 ▶); molecular graphics: *PLATON* (Spek, 2009 ▶); software used to prepare material for publication: *SHELXL97*.

## Supplementary Material

Crystal structure: contains datablock(s) I, global. DOI: 10.1107/S1600536812003017/im2350sup1.cif

Structure factors: contains datablock(s) I. DOI: 10.1107/S1600536812003017/im2350Isup2.hkl

Supplementary material file. DOI: 10.1107/S1600536812003017/im2350Isup3.cml

Additional supplementary materials:  crystallographic information; 3D view; checkCIF report

## Figures and Tables

**Table 1:** Hydrogen-bond geometry (Å, °)

*D*—H⋯*A*	*D*—H	H⋯*A*	*D*⋯*A*	*D*—H⋯*A*
N2—H2⋯N3^i^	0.86	2.01	2.845 (5)	165
N4—H4⋯N1	0.86	2.02	2.848 (6)	162

Symmetry code: (i) 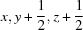.

**Fig. 1.**
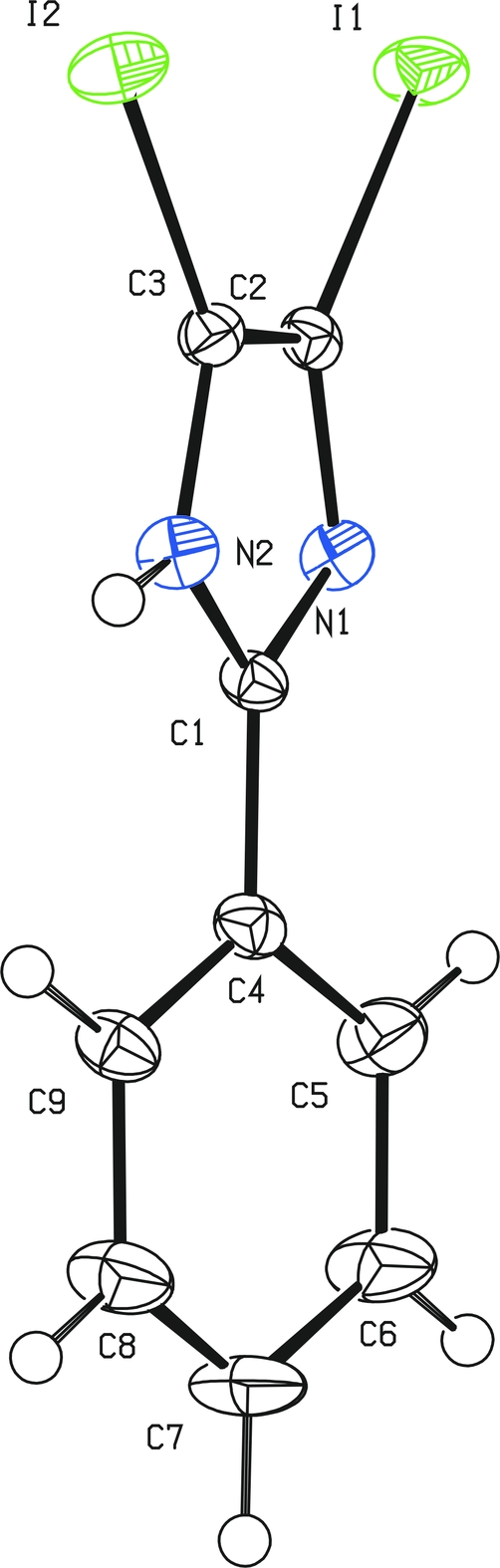
View of one of the independent molecules of the title compound with the displacement ellipsoids shown at the 50% probability level. H atoms are shown with arbitrary radii.

**Fig. 2.**
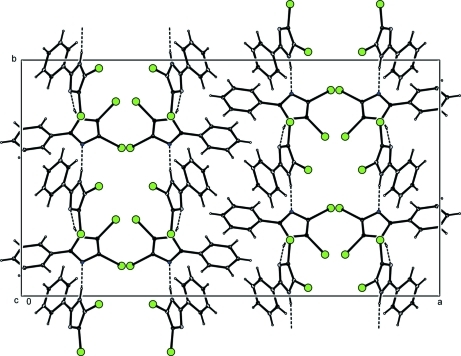
View of the crystal structure down the c axis showing the hydrogen bond interactions.

## supplementary crystallographic information

### Comment

Halogenated derivatives of heterocycles are important starting compounds for building new heterocyclic chiral ligands via cross-coupling reactions. In recent times 4,5-diiodoimidazole and its derivatives were used for the preparation of various disubstituted 4,5-dicarbaimidazoles (Zhang *et al*., 2006; Ito & Uedaira, 2004; Kim *et al.*, 1999; Haruki *et al.*, 1965). To the best of our knowledge, there are a couple of structures of dihalogenated imidazoles determined by X-ray diffraction methods, but the 4,5-diido-1*H*-imidazole structure as the most versatile and simpliest member of the series is missing. Compound I (Scheme 1) was separated as a byproduct from a reaction described by Ishihara (Ishihara & Togo, 2006) but is usually prepared by the method of Garden (Garden *et al.*, 2001). The title compound crystallizes (Fig. 1) in an orthorhombic space group with two independent molecules in the unit cell (Fig. 2). Both molecules reveal similar structural behavior showing two planar conjugated rings – phenyl and imidazolyl. The interplanar angles between imidazolyl and phenyl planes are 16.35 (3)° and 17.48 (6)°, respectively. The formation of infinite chains is provided by the connection of both types of molecules via N–H···N hydrogen bridges. The linear chains interact via the iodine atoms at neighbouring imidazole moieties. In contrast to typical C–I bond lenghts of ca. 2.095 Å (Allen *et al.*, 1987) which is the same for monoiodoimidazoles, the C–I separations in diiodoimidazoles significantly differ between ca. 2.05 and 2.09 Å (Panday *et al.*, 2000; Terinek & Vasella, 2003). This phenomenon is also seen in one of the molecules of I, where the C11–I3 bond length is 2.075 (5) Å and the C12–I4 bond located next to the N–H function is shortened to 2.058 (5) Å. On the other hand, in the first molecule both C–I bond lenghts (2.051 (5) and 2.058 (5) Å) are almost identical and thus comparable to the same parameters found in compounds containing the 4,5-diidoimidazolium ion (Mukai & Nishikawa, 2010*a*,*b*). Only one iodine atom I1 is located in the same plane defined by the imidazole ring, while the others show deviations of 0.07-0.148 Å. Bond lenghts between the atoms forming the imidazole rings are comparable to literature values (Allen *et al.*, 1987) except of C1–N2 which is elongated by 0.02 Å and C10–N3 which is slightly shortened.

### Experimental

The title compound I was isolated in 30% yield as a byproduct from the reaction mixture of 2-phenyl-1*H*-imidazole-4-carbaldehyde with ethan-1,2-diamine, iodine and potassium carbonate in tert-butylalcohol, according to Ishihara (Ishihara & Togo, 2006) in order to prepare 2'-phenyl-4,5-dihydro-1*H*,1'*H*-2,4'-biimidazole. The reaction mixture was separated by the help of column chromatography on silicagel (ethyl acetate, hexane and dichloromethane – 1:3:10). Further crystallization from dichloromethane gave pure I. The identity and purity of I was confirmed by the same melting point, ^1^H NMR and mass spectra patterns as published elsewhere (Garden *et al.*, 2001). Single crystals of I were obtained by slow vapour diffussion of hexane into a solution of I in dichloromethane.

### Refinement

All hydrogen atoms were discernible in the difference electron density map. However, all hydrogen atoms were placed into idealized positions and refined riding on their parent C or N atoms, with N–H = 0.86 Å, C–H = 0.93 Å for aromatic H atoms, with U(H) = 1.2U*_eq_*(C/N) for the NH group and U(H) = 1.5U*_eq_*(C/N) for other H atoms, respectively.

### Crystal data

**Table:**

C_9_H_6_I_2_N_2_	*D*_x_ = 2.409 Mg m^−^^3^
*M**_r_* = 395.96	Melting point: 472 K
Orthorhombic, *A**b**a*2	Mo *K*α radiation, λ = 0.71073 Å
Hall symbol: A 2 -2ac	Cell parameters from 20579 reflections
*a* = 31.0150 (6) Å	θ = 1–27.5°
*b* = 17.5010 (5) Å	µ = 5.72 mm^−^^1^
*c* = 8.0461 (9) Å	*T* = 150 K
*V* = 4367.4 (5) Å^3^	Needle, colourless
*Z* = 16	0.45 × 0.16 × 0.07 mm
*F*(000) = 2880	

### Data collection

**Table:**

Bruker–Nonius KappaCCD area-detector diffractometer	4914 independent reflections
Radiation source: fine-focus sealed tube	4499 reflections with *I* > 2σ(*I*)
graphite	*R*_int_ = 0.058
Detector resolution: 9.091 pixels mm^-1^	θ_max_ = 27.5°, θ_min_ = 2.3°
φ and ω scans to fill the Ewald sphere	*h* = −39→40
Absorption correction: gaussian (Coppens, 1970)	*k* = −22→19
*T*_min_ = 0.256, *T*_max_ = 0.675	*l* = −9→10
20468 measured reflections	

### Refinement

**Table:**

Refinement on *F*^2^	Secondary atom site location: difference Fourier map
Least-squares matrix: full	Hydrogen site location: inferred from neighbouring sites
*R*[*F*^2^ > 2σ(*F*^2^)] = 0.029	H-atom parameters constrained
*wR*(*F*^2^) = 0.051	*w* = 1/[σ^2^(*F*_o_^2^) + (0.0196*P*)^2^ + 8.5806*P*] where *P* = (*F*_o_^2^ + 2*F*_c_^2^)/3
*S* = 1.04	(Δ/σ)_max_ = 0.001
4914 reflections	Δρ_max_ = 0.63 e Å^−^^3^
235 parameters	Δρ_min_ = −0.63 e Å^−^^3^
1 restraint	Absolute structure: Flack (1983), 2233 Friedel pairs
Primary atom site location: structure-invariant direct methods	Flack parameter: 0.01 (3)

### Special details

**Table:**

Geometry. All esds (except the esd in the dihedral angle between two l.s. planes) are estimated using the full covariance matrix. The cell esds are taken into account individually in the estimation of esds in distances, angles and torsion angles; correlations between esds in cell parameters are only used when they are defined by crystal symmetry. An approximate (isotropic) treatment of cell esds is used for estimating esds involving l.s. planes.
Refinement. Refinement of F^2^ against ALL reflections. The weighted R-factor wR and goodness of fit S are based on F^2^, conventional R-factors R are based on F, with F set to zero for negative F^2^. The threshold expression of F^2^ > 2sigma(F^2^) is used only for calculating R-factors(gt) etc. and is not relevant to the choice of reflections for refinement. R-factors based on F^2^ are statistically about twice as large as those based on F, and R- factors based on ALL data will be even larger.

### Fractional atomic coordinates and isotropic or equivalent isotropic displacement parameters (Å^2^)

**Table:**

	*x*	*y*	*z*	*U*_iso_*/*U*_eq_	
I3	0.239603 (10)	0.127609 (18)	0.00147 (5)	0.02406 (8)	
I2	0.185474 (11)	0.465127 (18)	0.86344 (5)	0.02557 (9)	
I1	0.142369 (12)	0.262855 (18)	0.73764 (5)	0.02639 (9)	
I4	0.225974 (10)	0.324363 (17)	0.26443 (4)	0.02035 (8)	
N1	0.11736 (13)	0.3687 (2)	0.4558 (5)	0.0172 (9)	
N3	0.14573 (13)	0.1439 (2)	0.0814 (5)	0.0160 (8)	
C14	0.05141 (17)	0.1305 (3)	0.0519 (7)	0.0210 (12)	
H14	0.0683	0.1040	−0.0242	0.025*	
C9	0.11213 (17)	0.5450 (3)	0.2022 (6)	0.0217 (12)	
H9	0.1321	0.5741	0.2610	0.026*	
C3	0.15163 (16)	0.4339 (3)	0.6553 (6)	0.0166 (10)	
C1	0.11925 (16)	0.4415 (3)	0.4126 (6)	0.0153 (10)	
C4	0.10087 (15)	0.4731 (2)	0.2585 (7)	0.0178 (10)	
C13	0.07108 (16)	0.1796 (3)	0.1643 (6)	0.0151 (10)	
C11	0.18543 (15)	0.1775 (3)	0.1070 (6)	0.0163 (10)	
C2	0.13758 (15)	0.3637 (3)	0.6074 (6)	0.0146 (10)	
C12	0.18168 (16)	0.2429 (3)	0.1949 (6)	0.0170 (10)	
C10	0.11796 (16)	0.1907 (3)	0.1591 (6)	0.0150 (10)	
N2	0.13961 (13)	0.4833 (2)	0.5321 (5)	0.0184 (9)	
H2	0.1441	0.5318	0.5297	0.022*	
C18	0.04545 (16)	0.2180 (3)	0.2794 (7)	0.0219 (11)	
H18	0.0582	0.2511	0.3556	0.026*	
C5	0.07161 (18)	0.4297 (3)	0.1697 (7)	0.0269 (13)	
H5	0.0637	0.3815	0.2079	0.032*	
C16	−0.01789 (19)	0.1579 (3)	0.1692 (8)	0.0301 (14)	
H16	−0.0476	0.1507	0.1714	0.036*	
N4	0.13889 (12)	0.2501 (2)	0.2280 (6)	0.0147 (8)	
H4	0.1273	0.2867	0.2834	0.018*	
C17	0.00127 (17)	0.2077 (3)	0.2786 (8)	0.0296 (13)	
H17	−0.0156	0.2343	0.3548	0.035*	
C15	0.00750 (18)	0.1195 (3)	0.0545 (8)	0.0284 (13)	
H15	−0.0053	0.0865	−0.0215	0.034*	
C8	0.0937 (2)	0.5739 (3)	0.0577 (7)	0.0313 (14)	
H8	0.1005	0.6230	0.0224	0.038*	
C7	0.0656 (2)	0.5299 (3)	−0.0314 (8)	0.0343 (15)	
H7	0.0542	0.5487	−0.1302	0.041*	
C6	0.0541 (2)	0.4581 (3)	0.0261 (8)	0.0347 (15)	
H6	0.0350	0.4285	−0.0352	0.042*	

### Atomic displacement parameters (Å^2^)

**Table:**

	*U*^11^	*U*^22^	*U*^33^	*U*^12^	*U*^13^	*U*^23^
I3	0.02447 (18)	0.02458 (16)	0.02313 (18)	0.00572 (12)	0.00691 (17)	−0.00173 (16)
I2	0.03117 (19)	0.02441 (17)	0.02114 (18)	0.00247 (14)	−0.01031 (16)	−0.00504 (16)
I1	0.0322 (2)	0.01704 (15)	0.0299 (2)	−0.00235 (14)	−0.00951 (16)	0.00849 (16)
I4	0.01828 (16)	0.01997 (15)	0.02279 (17)	−0.00410 (12)	−0.00100 (14)	−0.00314 (15)
N1	0.019 (2)	0.012 (2)	0.020 (2)	0.0002 (16)	−0.0024 (17)	−0.0020 (17)
N3	0.022 (2)	0.0113 (19)	0.014 (2)	0.0012 (16)	−0.0021 (17)	−0.0004 (17)
C14	0.023 (3)	0.019 (3)	0.021 (3)	0.003 (2)	−0.003 (2)	−0.002 (2)
C9	0.030 (3)	0.018 (3)	0.017 (3)	−0.003 (2)	−0.001 (2)	0.000 (2)
C3	0.016 (2)	0.017 (2)	0.017 (3)	−0.0009 (19)	−0.002 (2)	0.001 (2)
C1	0.019 (3)	0.013 (2)	0.014 (3)	−0.0019 (19)	−0.0007 (19)	0.0012 (19)
C4	0.020 (2)	0.019 (2)	0.014 (3)	0.0032 (19)	0.002 (2)	0.000 (2)
C13	0.021 (3)	0.011 (2)	0.014 (3)	0.0038 (18)	−0.006 (2)	0.0051 (19)
C11	0.019 (2)	0.013 (2)	0.017 (3)	0.0060 (18)	0.004 (2)	−0.001 (2)
C2	0.015 (2)	0.015 (2)	0.014 (3)	0.0024 (18)	0.003 (2)	0.0008 (19)
C12	0.018 (3)	0.014 (2)	0.019 (3)	−0.0025 (19)	0.001 (2)	0.001 (2)
C10	0.024 (3)	0.010 (2)	0.012 (2)	−0.0031 (18)	−0.001 (2)	−0.0004 (18)
N2	0.024 (2)	0.0103 (19)	0.021 (3)	0.0011 (16)	−0.0028 (18)	−0.0033 (17)
C18	0.022 (3)	0.018 (2)	0.027 (3)	0.0016 (19)	−0.001 (2)	−0.001 (2)
C5	0.028 (3)	0.019 (3)	0.034 (3)	−0.003 (2)	−0.008 (2)	0.005 (2)
C16	0.020 (3)	0.037 (3)	0.033 (4)	−0.008 (2)	−0.002 (3)	0.001 (3)
N4	0.017 (2)	0.0127 (18)	0.014 (2)	0.0015 (15)	−0.0053 (17)	−0.0043 (17)
C17	0.017 (3)	0.034 (3)	0.038 (4)	−0.001 (2)	0.002 (3)	0.002 (3)
C15	0.023 (3)	0.031 (3)	0.031 (3)	−0.006 (2)	−0.010 (2)	0.000 (2)
C8	0.045 (4)	0.024 (3)	0.026 (3)	−0.006 (2)	−0.009 (3)	0.007 (2)
C7	0.047 (4)	0.030 (3)	0.026 (4)	0.003 (3)	−0.015 (3)	0.009 (3)
C6	0.044 (4)	0.030 (3)	0.030 (4)	−0.013 (2)	−0.020 (3)	0.004 (3)

### Geometric parameters (Å, °)

**Table:**

I3—C11	2.075 (5)	C13—C10	1.467 (7)
I2—C3	2.051 (5)	C11—C12	1.350 (7)
I1—C2	2.058 (5)	C12—N4	1.360 (6)
I4—C12	2.058 (5)	C10—N4	1.345 (6)
N1—C1	1.323 (6)	N2—H2	0.8600
N1—C2	1.374 (6)	C18—C17	1.382 (7)
N3—C10	1.343 (6)	C18—H18	0.9300
N3—C11	1.379 (6)	C5—C6	1.370 (8)
C14—C15	1.376 (8)	C5—H5	0.9302
C14—C13	1.388 (7)	C16—C17	1.374 (8)
C14—H14	0.9301	C16—C15	1.388 (9)
C9—C4	1.381 (7)	C16—H16	0.9299
C9—C8	1.391 (8)	N4—H4	0.8600
C9—H9	0.9301	C17—H17	0.9300
C3—C2	1.360 (7)	C15—H15	0.9299
C3—N2	1.366 (6)	C8—C7	1.367 (8)
C1—N2	1.363 (6)	C8—H8	0.9301
C1—C4	1.472 (7)	C7—C6	1.385 (8)
C4—C5	1.383 (7)	C7—H7	0.9299
C13—C18	1.393 (7)	C6—H6	0.9299
			
C1—N1—C2	105.9 (4)	N4—C10—C13	124.7 (4)
C10—N3—C11	104.1 (4)	C1—N2—C3	107.4 (4)
C15—C14—C13	120.8 (5)	C1—N2—H2	126.0
C15—C14—H14	119.8	C3—N2—H2	126.6
C13—C14—H14	119.3	C17—C18—C13	120.0 (5)
C4—C9—C8	120.1 (5)	C17—C18—H18	120.3
C4—C9—H9	119.9	C13—C18—H18	119.8
C8—C9—H9	120.0	C6—C5—C4	119.8 (5)
C2—C3—N2	106.2 (4)	C6—C5—H5	120.3
C2—C3—I2	129.5 (4)	C4—C5—H5	120.0
N2—C3—I2	124.3 (3)	C17—C16—C15	119.2 (5)
N1—C1—N2	110.6 (4)	C17—C16—H16	120.2
N1—C1—C4	124.5 (4)	C15—C16—H16	120.6
N2—C1—C4	124.9 (4)	C10—N4—C12	108.6 (4)
C9—C4—C5	119.8 (5)	C10—N4—H4	126.0
C9—C4—C1	121.4 (4)	C12—N4—H4	125.4
C5—C4—C1	118.9 (4)	C16—C17—C18	121.0 (5)
C14—C13—C18	118.7 (5)	C16—C17—H17	119.8
C14—C13—C10	119.9 (4)	C18—C17—H17	119.2
C18—C13—C10	121.4 (4)	C14—C15—C16	120.2 (5)
C12—C11—N3	111.2 (4)	C14—C15—H15	120.0
C12—C11—I3	129.8 (4)	C16—C15—H15	119.7
N3—C11—I3	118.9 (3)	C7—C8—C9	119.8 (5)
C3—C2—N1	109.9 (4)	C7—C8—H8	120.4
C3—C2—I1	127.4 (4)	C9—C8—H8	119.9
N1—C2—I1	122.6 (3)	C8—C7—C6	119.9 (5)
C11—C12—N4	105.4 (4)	C8—C7—H7	119.4
C11—C12—I4	132.2 (4)	C6—C7—H7	120.6
N4—C12—I4	122.3 (3)	C5—C6—C7	120.6 (5)
N3—C10—N4	110.7 (4)	C5—C6—H6	119.9
N3—C10—C13	124.6 (4)	C7—C6—H6	119.4

### Hydrogen-bond geometry (Å, °)

**Table:**

*D*—H···*A*	*D*—H	H···*A*	*D*···*A*	*D*—H···*A*
N2—H2···N3^i^	0.86	2.01	2.845 (5)	165
N4—H4···N1	0.86	2.02	2.848 (6)	162

Symmetry codes: (i) *x*, *y*+1/2, *z*+1/2.
